# One-dimensional CuO nanowire: synthesis, electrical, and optoelectronic devices application

**DOI:** 10.1186/1556-276X-9-637

**Published:** 2014-11-26

**Authors:** Lin-Bao Luo, Xian-He Wang, Chao Xie, Zhong-Jun Li, Rui Lu, Xiao-Bao Yang, Jian Lu

**Affiliations:** 1 School of Electronic Science and Applied Physics, Hefei University of Technology, Hefei, Anhui 230009, People’s Republic of China; 2 Department of Mechanical and Biomedical Engineering, City University of Hong Kong, Kowloon, Hong Kong SAR, People’s Republic of China; 3 Department of Physics, South China University of Technology, Guangzhou, Guangdong Province 510641, People’s Republic of China; 4 Centre for Advanced Structural Materials, City University of Hong Kong Shenzhen Research Institute, 8 Yuexing 1st Road, Shenzhen Hi-Tech Industrial Park, Shenzhen, People’s Republic of China

**Keywords:** Surface mechanical attrition treatment (SMAT), Semiconductor nanostructures, The first-principle calculation, Metal oxide, Flexible photodetector

## Abstract

In this work, we presented a surface mechanical attrition treatment (SMAT)-assisted approach to the synthesis of one-dimensional copper oxide nanowires (CuO NWs) for nanodevices applications. The as-prepared CuO NWs have diameter and the length of 50 ~ 200 nm and 5 ~ 20 μm, respectively, with a preferential growth orientation along [1 $\bar{1}$ 0] direction. Interestingly, nanofield-effect transistor (nanoFET) based on individual CuO NW exhibited typical *p*-type electrical conduction, with a hole mobility of 0.129 cm^2^V^-1^ s^-1^ and hole concentration of 1.34 × 10^18^ cm^-3^, respectively. According to first-principle calculations, such a *p*-type electrical conduction behavior was related to the oxygen vacancies in CuO NWs. What is more, the CuO NW device was sensitive to visible light illumination with peak sensitivity at 600 nm. The responsitivity, conductive gain, and detectivity are estimated to be 2.0 × 10^2^ A W^-1^, 3.95 × 10^2^ and 6.38 × 10^11^ cm Hz^1/2^ W^-1^, respectively, which are better than the devices composed of other materials. Further study showed that nanophotodetectors assembled on flexible polyethylene terephthalate (PET) substrate can work under different bending conditions with good reproducibility. The totality of the above results suggests that the present CuO NWs are potential building blocks for assembling high-performance optoelectronic devices.

## Background

Metal oxide semiconductors (e.g. ZnO, [1] TiO_2_, [2] NiO, [3] SnO_2_[4], and CuO [5]) are one of the most common, most diverse and probably the richest class of materials among the various groups of semiconductors. In the past decade, a number of methods including laser ablation [6,7], thermal oxidation [8,9], solution-phase growth [10], and template-assisted synthesis [11] have been employed to fabricate various one-dimensional metal oxide semiconductor nanostructures, such as nanowires, nanotubes, and nanoribbons [12]. Due to the high surface-volume ratio and quantum-size effect, the resultant nanostructures with improved physical, optical, and electronic properties [13] have been used as building blocks to construct a number of optoelectronic and electronic devices including solar cells [14,15], photodetectors [16,17], gas sensors [18], non-volatile memory devices [19], and so on.

Copper oxide (CuO), as one of the most important metal oxide semiconductors, has been widely used because of its abundance in resources and low cost in synthesis. Low-dimensional CuO nanostructures (zero-dimensional and one-dimensional nanostructures) are used, in particular *via* simple thermal evaporation method [20], wet chemical method [21], and metal-assisted growth method [22]. It has been found that the CuO NWs obtained from the above methods normally have good crystallinity and high aspect ratio, which renders them attractive and promising building blocks for fabricating high-performance electronic devices systems [23]. For example, Chang et al. reported the growth of CuO NWs on an oxidized Cu wire at 500°C for infrared (IR) photodetection application. The as-obtained high density of CuO NWs on the Cu wire was highly sensitive to IR light illumination (wavelength: 808 nm), with rise-time and fall-time of 15 and 17 s, respectively [24]. Zhou et al. presented a vertically aligned CuO NWs array-based ultrasensitive sensors for H_2_S detection with a detection limit as low as 500 ppb. It was revealed that the high sensitivity was due to the formation of highly conductive CuS layer when H_2_S gas was introduced into the detection chamber [25]. Zheng et al. developed a simple and effective catalyst system comprised of CuO NWs for CO oxidation. They found that CO oxidation percentage was as high as 85% after Ar or H_2_ plasma treatment [5]. In addition to these device applications, it has been observed that highly-aligned CuO NW arrays are good candidates for field emission due to their low turn-on voltages, high current output [26].

Despite of the above research progresses, there is a sparsity of research activity dealing with the transport and optoelectronic property of individual CuO nanostructures [27], which constitutes the basic building blocks of various optoelectronic and electronic devices. Exploration along this direction is highly desirable as it is not only helpful for understanding the electrical property of individual CuO NWs, but also beneficial to the development of high-performance optoelectronic and electronic devices. Herein, we report the synthesis of CuO NWs by heating surface mechanical attrition treatment (SMAT) processed copper foil in tube furnace. The CuO NW is of single crystal with a growth direction of [1 $\bar{1}$ 0]. Individual CuO NW-based field-effect transistor displays weak *p*-type electrical conduction behavior, which was probably due to the O defects, according to the theoretical simulation based on first-principle calculation. Further optoelectronic characterization shows that the CuO NW is sensitive to incident light of 600 nm, with high producibility and stability. It is also observed that the photodetector fabricated on flexible polyethylene terephthalate (PET) substrate showed good reproducibility under different bending conditions. The above result suggests that our CuO NWs will have promising potential in future devices applications.

## Methods

### Synthesis and structural characterization of the CuO NWs

In this study, the CuO NWs were fabricated via SMAT-assisted thermal oxidation method. Briefly, copper plates (99.99%) with size of 20 × 20 × 5 mm were cleaned by alcohol to remove surface impurities including grease and other organics. The copper plates were then treated by an SMAT process in which millimeter-size steel balls were acoustically driven to bombard the Cu surface randomly and in all directions to generate nanocrystalline Cu [28]. After drying in N_2_ atmosphere, the clean samples were heated in a horizontal tube at 500°C in pure O_2_ atmosphere (375 Torr) for 2.5 h. The morphologies and structure of the as-prepared CuO NWs were characterized by scanning electron microscopy (SEM, FEI Quanta 200 FEG, FEI, Hillsboro, OR, USA), energy-dispersive X-ray spectroscopy (EDS), high-resolution transmission electron microscopy (HRTEM, JEOL JEM-2010 at 200 kV, JEOL, Akishima-shi, Tokyo, Japan), X-ray diffraction (XRD, Rigaku D/Max-γB, with Cu Kα radiation, Rigaku Corporation, Tokyo, Japan) and X-ray photoelectron spectroscopy (XPS, ThermoESCALAB250, Thermo Fisher Scientific, Waltham, MA, USA).

### Device fabrication and characterization

To evaluate the electrical properties of the CuO NWs, back-gate field-effect transistor (FET) was constructed based on individual CuO NW. Firstly, the as-synthesized CuO NW was dispersed on a SiO_2_/*p*^*+*^-Si substrate by a contact print technique [29], then Cu (4 nm)/Au (50 nm) source and drain electrodes were defined by photolithography and e-beam evaporation. In order to achieve ohmic contact between the NW and electrodes, the as-fabricated devices were annealed at 200°C for 10 min in argon atmosphere at a pressure of 0.33 Torr. In this work, flexible photodetectors on PET substrate were constructed by the same process. Both the electrical and optoelectronic characterization of CuO NW-based devices were carried out by using a semiconductor characterization system (Keithley 4200-SCS, Keithley, Cleveland, OH, USA).

### Theoretical simulation

The first-principle calculation of [$1\bar{1}$ 0] CuO NW were based on the density functional theory (DFT) implemented in the Vienna *ab initio* simulation package method [30,31]. The projector-augmented wave (PAW) [32] and the Perdew-Burke-Ernzerhof GGA (PBE) [33] functionals were employed for the total energy calculations. The cutoff energy was 450 eV and the criteria of the forces were set to be 0.01 eV/Å for all atoms. An 11 × 11 × 11 k-grid mesh was used for the bulk CuO and a 7× 1 × 1 mesh for the < $1\bar{1}$ 0 > CuO NW, where the vacuum distance was set to be 10 Å to avoid cell-to-cell interactions. To improve the calculations of electronic properties, we used the GGA + U extension to the DFT calculation [34,35], dealing with the Cu 3d electrons for a better description, where U = 7.5 eV and J = 1.0 eV were adopted.

## Results and discussion

**Figure 1:**
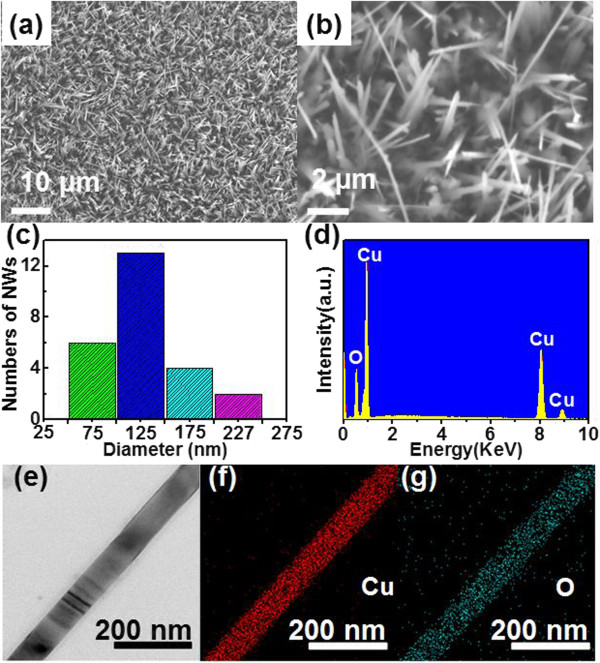
**SEM and TEM image, diameter distribution, and EDS spectrum of CuO NWs and Cu/O images. (a, b)** Typical SEM image of the CuO NWs at different magnifications; **(c)** statistical distribution of the diameter of CuO NWs; **(d)** the EDS spectrum of CuO NWs; **(e)** TEM image of a single CuO NW; elemental mapping images of Cu **(f)**, and O **(g)**.

The fabrication of the CuO NWs was carried out in a tube furnace in oxygen atmosphere. Figure 1a, b displays typical SEM images of as-synthesized CuO NWs with different magnifications. It is obvious that the product is composed of fiber-like structures with length of about 5 ~ 20 μm. The statistical distribution in Figure 1c shows that the diameters of the CuO NWs are in range of 50 ~ 250 nm, with an average value of approximately 120 nm. According to the corresponding EDX spectrum of the CuO NWs in Figure 1d, the product is composed of Cu and O elements with a molar ratio of approximately 51:49, indicative of the presence of CuO, rather than Cu_2_O. Figure 1f, g shows the elemental mapping image of an individual CuO NW, from which one can see that the constituting elements (Cu and O) are uniformly distributed in the nanostructure.

**Figure 2:**
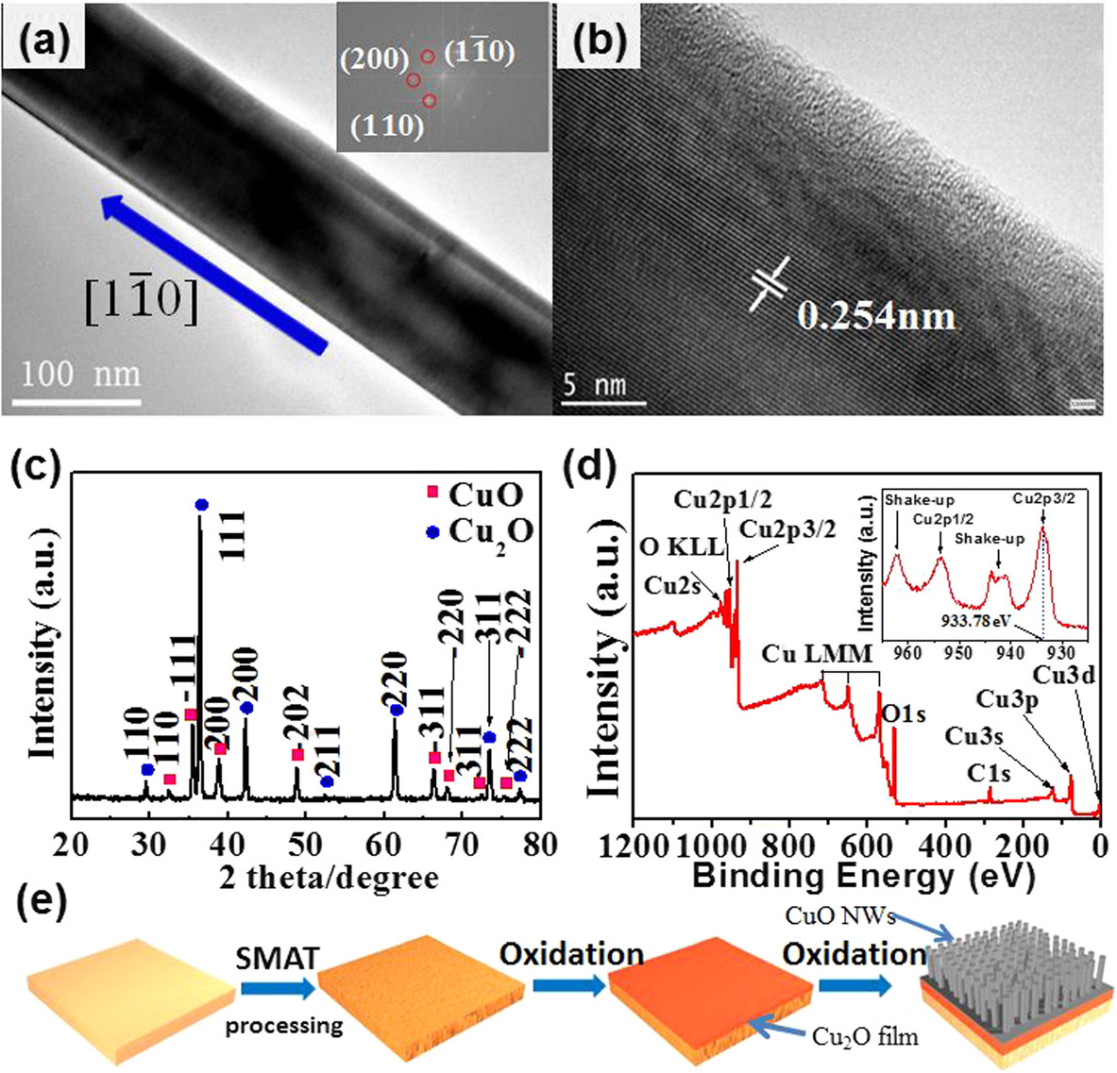
**TEM images, XRD pattern, XPS survey spectrum, and schematic illustration of CuO NWs. (a)** TEM image of the CuO NW, the inset shows the corresponding FFT pattern; **(b)** high-resolution TEM image of a CuO NW; **(c)** the XRD pattern of the CuO NW; **(d)** XPS survey spectrum of the CuO NW, the inset shows the corresponding high-resolution N1s spectrum; **(e)** schematic illustration of the formation of CuO NWs.

The TEM image in Figure 2a indicated that the surface of the NWs was free of impurities and contaminants. Further HRTEM image along with the corresponding fast Fourier transform (FFT) pattern in the inset of Figure 2a shows that the CuO NWs are of single crystal with preferential growth orientation along [1 $\bar{1}$ 0] direction. Figure 2c displays a typical XRD pattern of the product, in which the peaks labeled with red quadrate can be readily indexed to the monoclinic phase of CuO (JCPDS-80-1916) [4,11,20]. In fact, the presence of CuO was verified by the XPS analysis. As is shown in Figure 2d, two peaks of Cu 2p are located at 933.8 and 953.8 eV which represent the Cu 2p3/2 and Cu 2p1/2, respectively. These signals can be ascribed to the Cu 2p in CuO, in consistence with literature result. What is more, the strong shake-up satellites located at 940.92 and 943.83 eV confirm the presence of the Cu (II) valence state [6]. In addition, strong peaks (labeled with blue balls) ascribable to Cu_2_O phase were present in the pattern (JCPDS-05-0667) as well. We attribute the presence of Cu_2_O to the special growth mechanism, as illustrated in Figure 2e. At initial growth stage, Cu_2_O thin film was formed when the copper plate was treated with high temperature in the oxygen atmosphere. According to the previous study, in the underlying Cu_2_O layer, the CuO NWs are formed as a result of rapid, short-circuit diffusion of the Cu ions across grain boundaries and/or defects [28,36]. Notably, during this growth process, the SMAT processing is highly advantageous in that the generation of many dislocations, twins, or stacking faults in the surface of Cu plate will increase the quality, as well as the length of the NWs.

**Figure 3:**
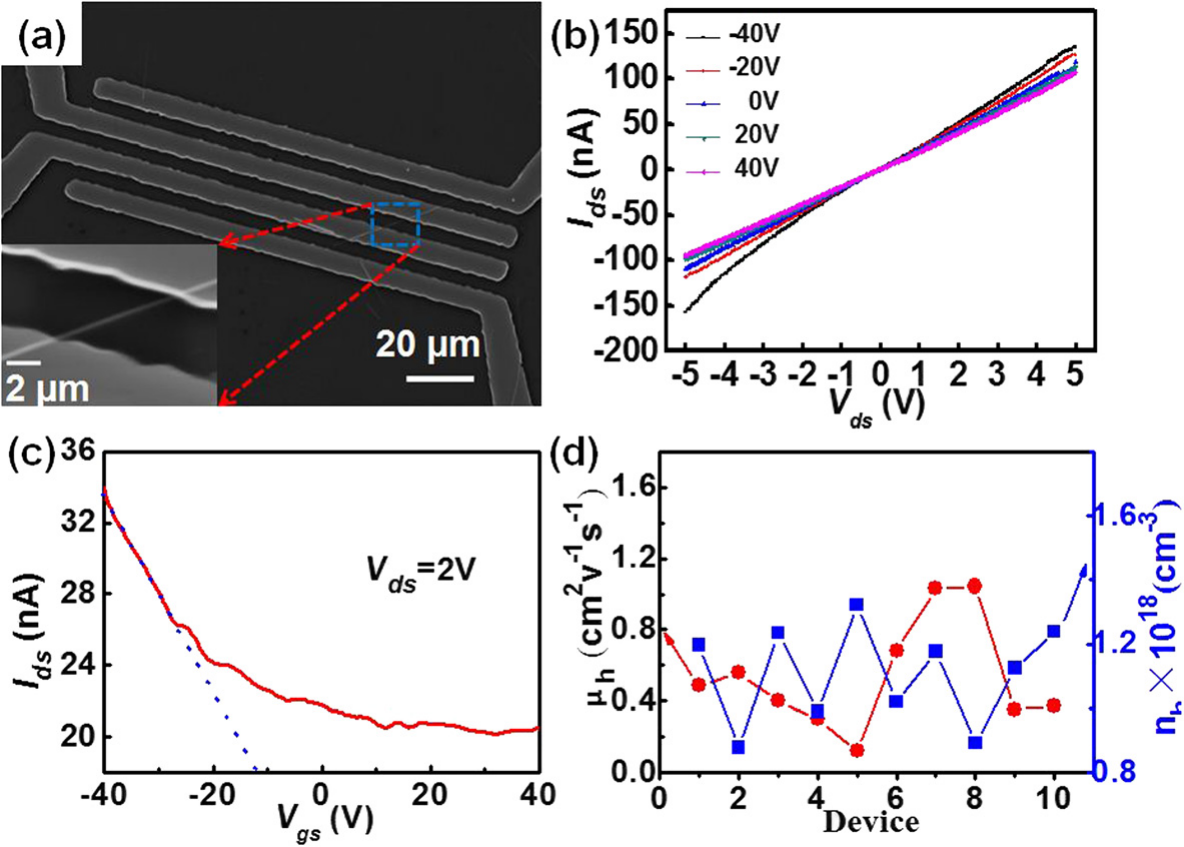
**SEM image of MOSFET device, I**_***ds***_**-V**_***ds ***_**curves, and characteristics of the nanoFETs. (a)** SEM image of the CuO NW MOSFET device, the inset shows the magnified image; **(b)***I*_*ds*_-*V*_*ds*_ curves at different gating voltages. **(c)** Transfer characteristics of the nanoFET at *V*_*ds*_ = 2 V. **(d)** Hole mobility and concentration of ten representative nanoFETs.

To study the transport properties of a single CuO NW, back-gate metal-oxide-semiconductor FETs (MOSFETs) were fabricated on the basis of individual CuO NW. The linear *I-V* curve in Figure 3a suggests that Cu (4 nm)/Au (50 nm) electrode can form good contact with relatively low contact barrier. Electrical study of the single CuO NW-based FET in Figure 3c exhibits typical *p*-type conduction behavior. That is, the electrical conduction increases with decreasing gate voltage. By fitting the linear part of the *I*_*ds*_-*V*_*g*_ characteristics, the turn-on threshold voltage (*V*_*T*_) and transconductance (gm = d*I*_*ds*_/d*V*_*g*_) are calculated to be -12 V and 0.54 nS, respectively. To evaluate the property of the CuO NW, two key parameters of hole mobility (*μ*_*h*_) and concentration (*n*_*h*_) were estimated. The hole mobility (*μ*_*h*_) and concentration (*n*_*h*_) can be calculated according to the following equations:

(1) $$\mu_{h}\;=\;g_{m}\frac{\mathrm{In}\left(4h/d\right)l}{2\pi \epsilon_{0}\epsilon_{SiO_{2}}V_{ds}}$$

(2) $$n_{h}=\frac{\sigma }{q{µ}_{h}}$$

Where *h, d,* and *l* represent the thickness of oxide layer (300 nm), the NW diameter (125 nm), and the channel length (5 μm), respectively. $\epsilon_{{SiO}_{2}}$ is the dielectric constant of the SiO_2_ dielectric layer (approximately 3.9), *ϵ*_0_ is the permittivity at vacuum, *σ* is the conductivity of the NW, and *q* is the charge of an electron. Based on the equation (Equation 1), the hole mobility is estimated to be 0.134 cm^2^V^-1^ s^-1^. Such a value is larger than the CuO thin film [37], and CuO NWs synthesized by direct evaporating Cu substrates in oxygen ambient without SMAT process [28], suggesting that the present SMAT-assisted thermal evaporation is an ideal approach to the synthesis of CuO NWs. Furthermore, the hole concentration is calculated to be 1.29 × 10^18^ cm^-3^ according to Equation 2. To obtain a statistical distribution of the CuO NWs, totally ten FETs were analyzed. As displayed in Figure 3d, the hole mobilities of most CuO NWs are in the range of 0.1 to 1.0 cm^2^V^-1^ s^-1^ with an average value of 0.58 cm^2^V^-1^ s^-1^. Meanwhile, the hole concentration is in the range of 0.8 × 10^18^ to 1.4 × 10^18^ cm^-3^ with an average value of 1.13 × 10^18^ cm^-3^.

**Figure 4:**
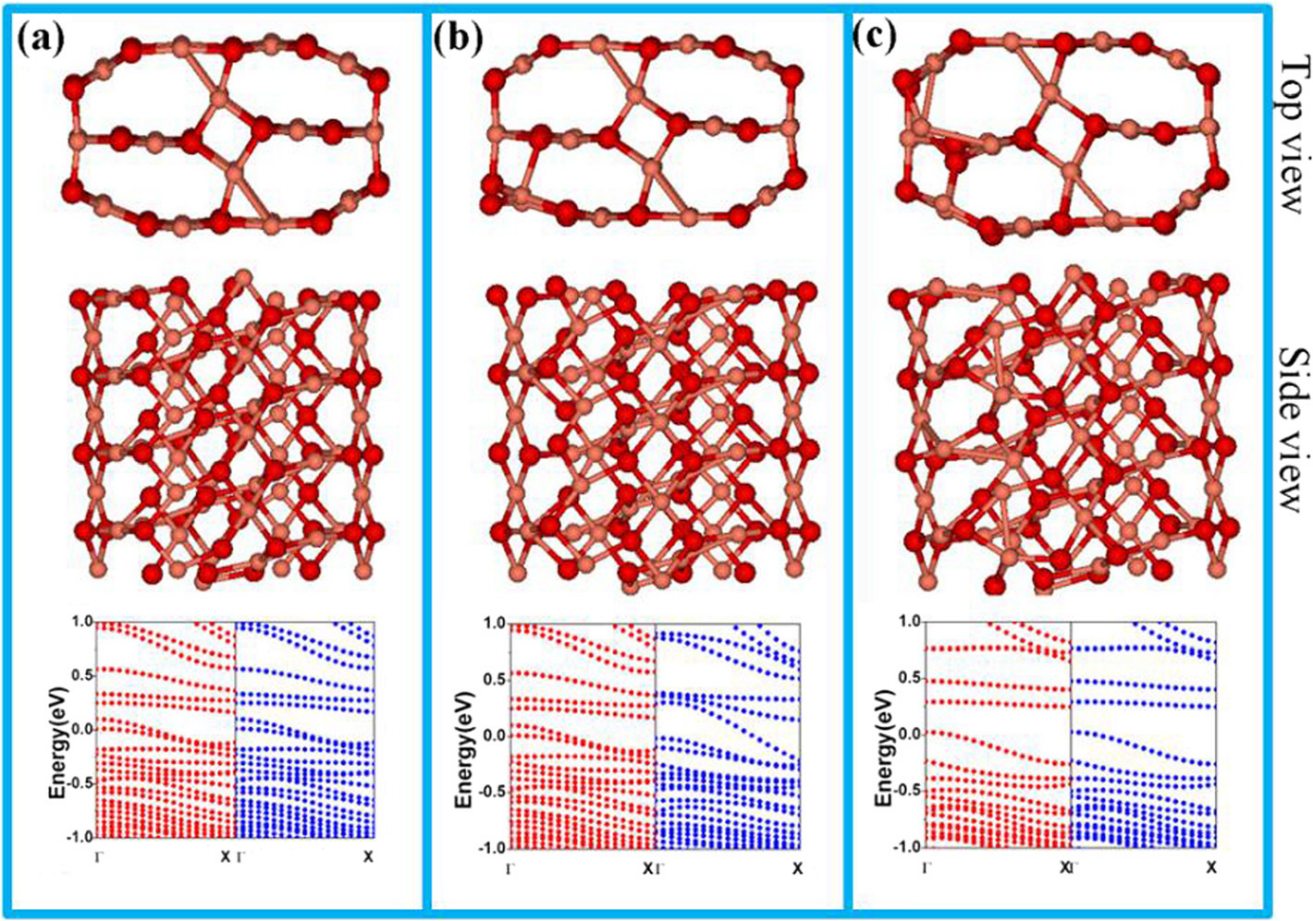
**Atomic configurations and band structures of CuO NW with and without defects. (a)** Ideal NW; **(b)** with a Cu vacancy; **(c)** with an O vacancy. Dark and light balls represent O and Cu atoms, respectively. Red/blue circles represent the band structures from electrons with spin up and down, respectively. The Fermi levels are shifted to zero.

**Figure 5:**
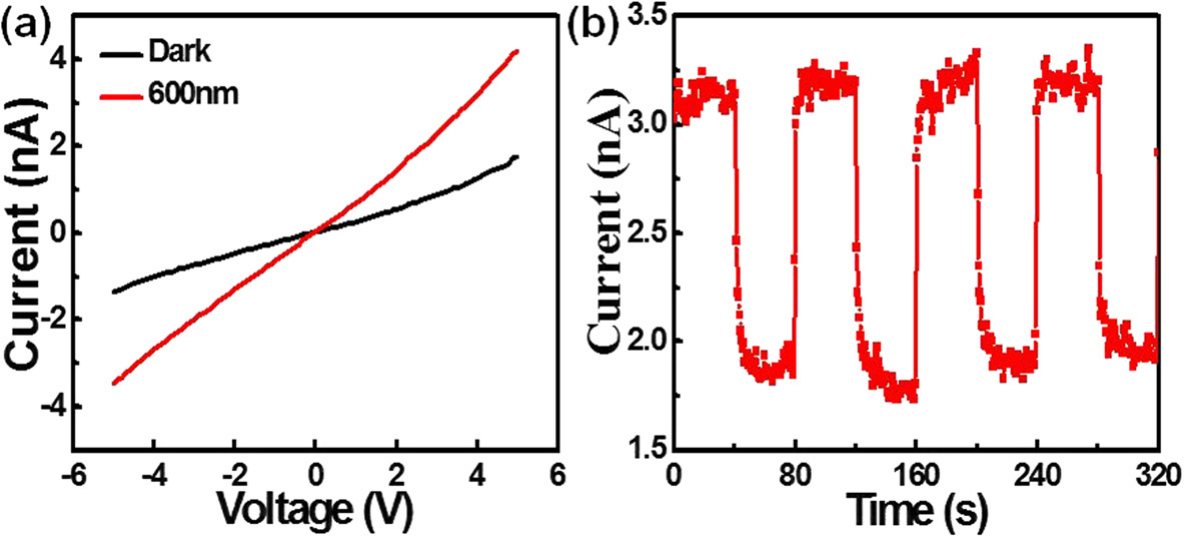
***I-V *characteristics of the CuO-based photodetector and time response spectra of the device. (a)***I-V* characteristics of the CuO-based photodetector measured at room temperature with and without light irradiation. **(b)** Time response spectra of the device when the incident light was manually switched on and off repeatedly (*V*_ds_ = 5 V).

To unveil the physical reason behind the *p*-type electrical characteristics, we used first-principle calculation to simulate the electronic structures of CuO NW with different surface defects. Firstly, we compared various possible magnetic states and found an anti-ferromagnetic ground state for bulk CuO, in agreement with the previous study [38]. Figure 4a shows the ground states of anti-ferromagnetic CuO NW, where Cu atoms exhibit local magnetic moments. Obviously, the bands near the Fermi level are not fully filled with electrons and thus the system should exhibit metallic characteristics for an ideal CuO NW. Similar phenomenon was also observed when there is a Cu vacancy in the crystal lattice, in which the corresponding band structures near the Fermi level are partially filled (shown in Figure 4b). These results suggest that neither CuO with a Cu vacancy nor ideal CuO without any defect can lead to the observed *p*-type conduction behavior. However, when an oxygen vacancy is present on the surface of CuO NW, the bands near the Fermi level are fully filled and there are two flat bands at around 0.25 ~ 0.50 eV, as is shown in Figure 4c. In this case, electrons can be readily stimulated and trapped in these flat bands, giving rise to *p*-type conducting characteristic. As a matter of fact, the presence of huge amount of surface defects was experimentally corroborated by the ESR spectrum as a function of external magnetic field shown in Additional file 1: Figure S1 of the supporting information.Next, the optoelectronic properties of the individual CuO NW photodetector (nano-PD) were studied. It is obvious that the CuO NW shows an obvious increase in current when the device is exposed to the 600-nm illumination (see Figure 5a). Additionally, the CuO NW device can be readily switched between low- and high-conduction states with relatively good reproducibility when the light illumination was turned on and off alternatively. In order to quantitatively evaluate the performance of the CuO NW-based device, three key parameters including responsivity (R), gain (G), and detectivity (D*) were calculated by the following equation:

(3) $$R\;\left(A\;\cdot \;W^{‒1}\right)\;=\;\frac{I_{l}\;-\;I_{d}}{P_{\text{opt}}}\;=\;\eta \left(\frac{q\lambda }{hc}\right)G$$

(4) $$G=N_{el}/N_{\mathrm{ph}}=\tau /\tau_{tr}$$

(5) $$D^{*}\;=\;A^{1/2}R/{\left(2qI_{d}\right)}^{1/2}$$

where *I*_*l*_ is the photocurrent, *I*_*d*_ is the dark current, *P*_opt_ is the incident light power, *η* is the quantum efficiency (we assume *η* = 1 for simplification), *q* is the charge of an electron, *λ* is the incident light wavelength, *h* is the Planck’s constant, and *c* is the speed of light. The values of *P*_opt_*q*, *λ*, *h*, *c* are 2 mW·cm^-2^, 1.6 × 10^-19^ C, 600 nm, 6.626 × 10^-34^ J·s, and 2.997 × 10^8^ m/s, respectively. Based on the equation (Equation 3), the responsitivity (R) of the device is estimated to be 2.0 × 10^2^ A·W^-1^ at the voltage of 5 V. Physically, the photoconductive gain (G) is defined as the ratio of the number of electrons collected per unit time (*N*_el_) and the number of absorbed photons per unit time (*N*_ph_), or equal to the ratio of carrier life time (τ) to carrier transit time (τ_tr_); it can be derived to be 3.95 × 10^2^ according to Equation 3 or Equation 4. By using Equation 5, the detectivity (D*) is estimated to be 6.38 × 10^11^ cm·Hz^1/2^ W^-1^ based on the above value and the active area (A) of 6.25 × 10^-9^ cm^2^ (effective area that absorbs the incident light). Table 1 summarizes the key metrics of the current device and other semiconductor nanostructure-based PDs. It is obvious that the R and G are comparable to the device based on pure CdTe NW [39], but much higher than that based on CdSe NW [40] and ZnO NW [41]. We believe this relatively good optoelectronic property is partially associated to the introduction of SMAT prior to oxidation which can improve the quality of the CuO NW during growth process.

**Table 1:** Summary of the device performances of the CuO-based PD with other PDs based on pure materials

**Materials**	**R/AW**^**-1**^	**G**	D*/cm · Hz^**1/2**^ **· W**^**-1**^	**Reference**
CuO NW	2.0 × 10^2^	3.95 × 10^2^	6.38 × 10^11^	Our work
CdTe NW	3.6 × 10^2^	5.56 × 10^2^	6.63 × 10^10^	[39]
CdSe NW	10 ~ 100	0.05	1.71 × 10^11^	[40]
ZnO	Approximately 0.055	Approximately 10^2^	7.43 × 10^11^	[41]

**Figure 6:**
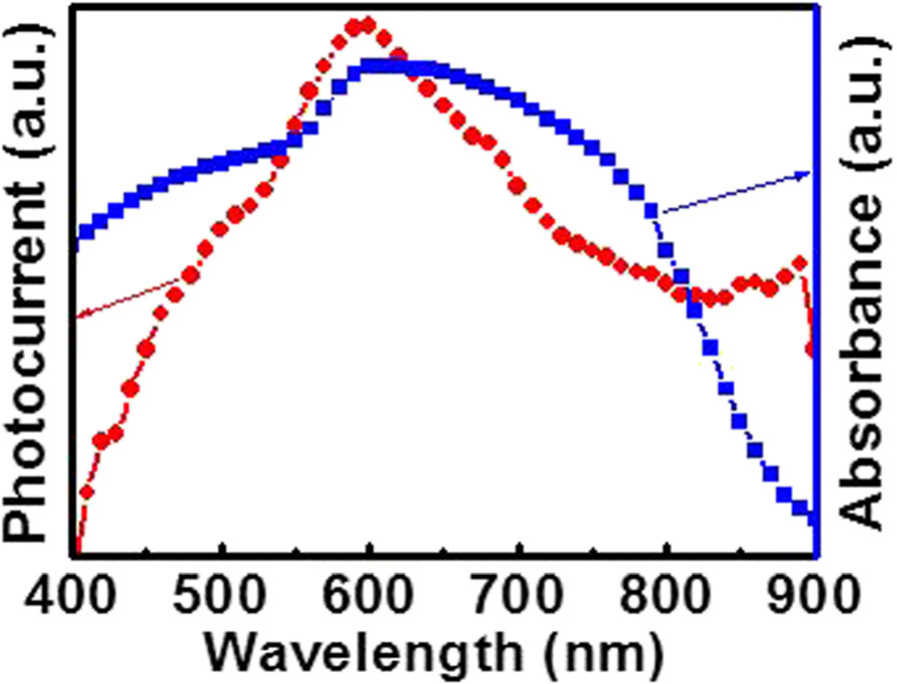
**UV–vis spectrum and spectral responses.** UV–vis spectrum of CuO NWs (blue line) and spectral response (red line) of individual CuO NW based nano-PD.

Figure 6 plots the UV–vis absorption of the CuO NW and the normalized responsitivity of the individual CuO NW-based nano-PD as a function of wavelength. To make the analysis more reliable, we kept the light power identical for all wavelengths during measurements. It is noted that the device exhibits high sensitivity to visible light, with sensitivity peak at 600 nm, in rough consistence with the cutting edge of UV–vis absorption curve (blue line). This agreement is believed to be highly related to the working mechanism of such photoconductive-type photodetector [17,42].

**Figure 7:**
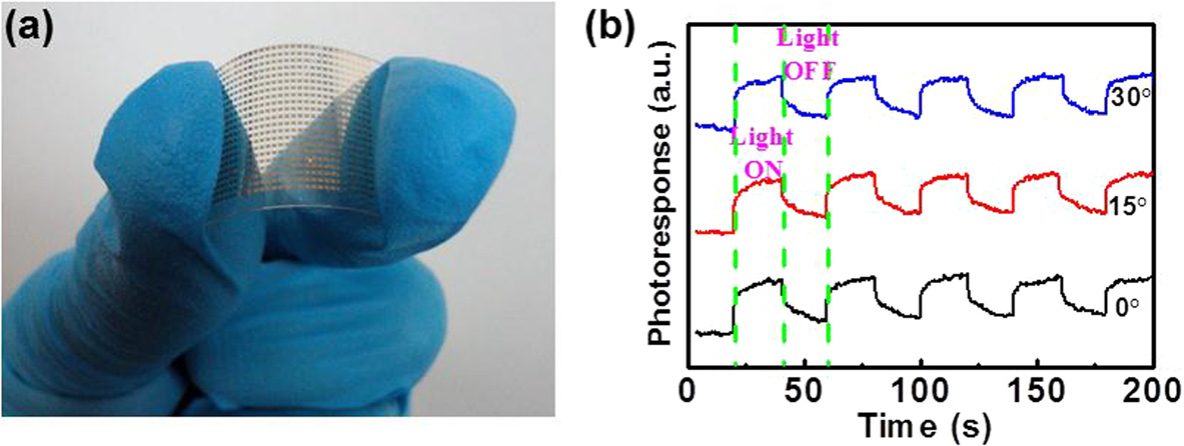
**Digital camera picture of and photoresponse of nano-PDs. (a)** Digital camera picture of nano-PDs fabricated on the flexible PET substrate; **(b)** photoresponse of the nano-PD on PET substrate with different bending angles: 0°, 15°, 30°.

Apart from nano-PDs on hard SiO_2_/Si substrate, nano-PDs were also fabricated by selecting flexible PET substrate. Figure 7a shows the device image under bending condition, from which one can see that the device exhibits excellent flexibility. Remarkably, the CuO NW device also displays obvious sensitivity to 600 nm under various bending conditions. Figure 7b compares the photoresponse of nano-PD after bending the PET substrate to different angles relative to the horizontal level. The *I*_on_/*I*_off_ is 1.35, 1.32, and 1.24 for angles of 0°, 15°, and 30°, respectively, suggesting that the current flexible CuO nano-PDs have great potential for the application in future transparent and flexible optoelectronics. In addition to the flexibility, the present devices are highly transparent in visible light range. As shown in Additional file 1: Figure S2, the transmittance is over 85% in the majority of the visible light range. This characteristic, combined with the flexibility and long-term stability (Additional file 1: Figure S3), makes the current device a good candidate for future optoelectronic device applications.

## Conclusions

In summary, we have fabricated one-dimensional CuO NW by heating SMAT copper plate in oxygen atmosphere. Electrical field-effect transistor device based on the as-prepared individual CuO NW exhibited typical *p*-type electrical conduction characteristic, with hole mobility and concentration of 0.134 cm^2^V^-1^ s^-1^ and 1.29 × 10^18^ cm^-3^, respectively. It is also revealed that the as-synthesized CuO NW was highly sensitive to light irradiation of 600 nm, with a high responsitivity and photoconductive gain of 2.0 × 10^2^ AW^-1^ and 3.95 × 10^2^, respectively. Further optoelectronic study shows that the photodetector on flexible PET substrate is also highly sensitive to 600-nm wavelength light at different bending conditions. The generality of this study proves that CuO NW obtained via SMAT-assisted thermal evaporation method will have great potential for future high-performance optoelectronic devices application.

## Competing interests

The authors declare that they have no competing interests.

## Author’s contributions

LBL, XHW, CX, and RL carried out the experiments. ZJL and XBY conducted the theoretical simulation. JL conceived the idea and supervised the whole work. LBL, XBY, and JL drafted the paper. All authors read and approved the final manuscript.

## Supplementary Material

Additional file 1 **The electron spin resonance (ESR) of the CuO NWs (Figure S1).** Transmittance of the CuO NWs device on flexible PET substrate (**Figure S2**). Comparison of photoresponse of the CuO NW before and after 1-month storage (**Figure S3**). Click here for file
