# Supplementary material for: One-dimensional CuO nanowire: synthesis, electrical, and optoelectronic devices application
Source: Nanoscale Res Lett. 2014 Nov 26;9(1):637. doi: 10.1186/1556-276X-9-637 (PMC4256975; doi:10.1186/1556-276X-9-637)
Supplement: Additional file 1 — The electron spin resonance (ESR) of the CuO NWs (Figure S1). Transmittance of the CuO NWs device on flexible PET substrate (Figure S2). Comparison of photoresponse of the CuO NW before and after 1-month storage (Figure S3). [file 1556-276X-9-637-S1.docx]

Supporting information

One dimensional CuO nanowire: synthesis, electrical and optoelectronic devices application

Lin-Bao Luo,^1, 2^ Xian-He Wang,^1^ Chao Xie,^1^ Zhong-Jun Li,^1^ Rui Lu,^1^ Xiao-Bao Yang, ^3*^ Jian Lu^2,4*^

***The Electron spin resonance (ESR) of the CuO NWs:***

γ(HZ)=9066.225

H(mT)=328.2725

g= 0.07145**×**γ(HZ)/H(mT)=1.9733


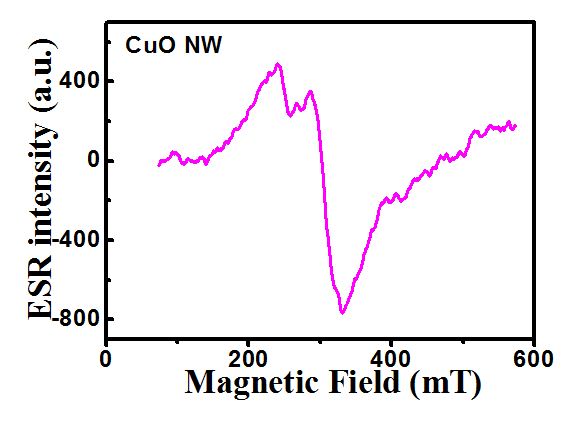


**SI-Figure 1.** ESR spectrum of the CuO NWs at room temperature.

**
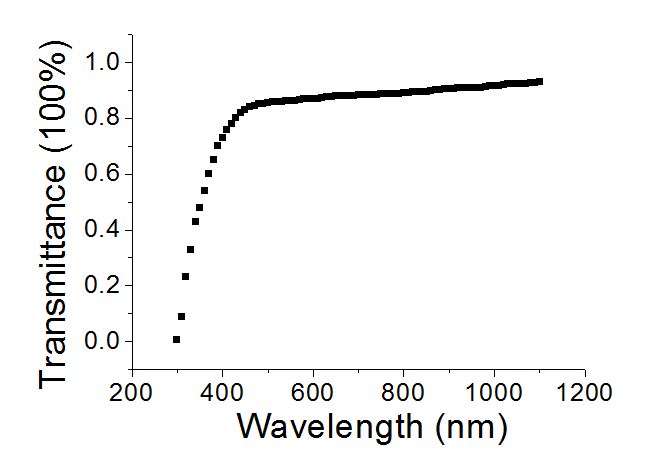
**

**SI-Figure 2.** Transmittance of the CuO NWs device on flexible PET substrate


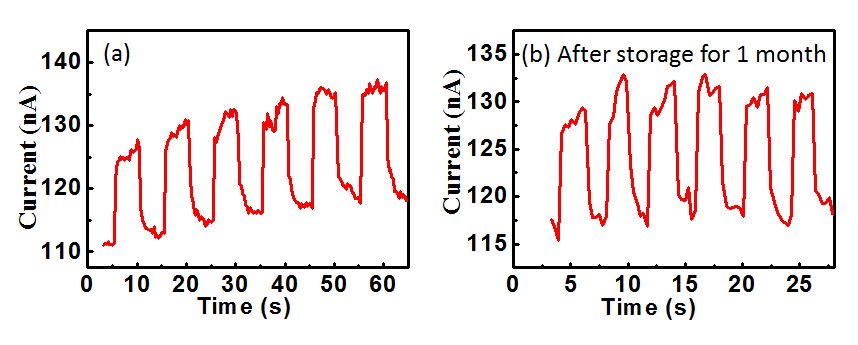


**SI-Figure 3.** Comparison of photoresponse of the CuO NW before and after 1 month storage.
